# Influence of e-Liquid pH on Heavy Metal Emissions in Open-System Electronic Cigarette Aerosols and Associated Health Risks

**DOI:** 10.1093/ntr/ntag016

**Published:** 2026-03-27

**Authors:** David Lawson, James Coulson

**Affiliations:** School of Medicine, Cardiff University, Cardiff, UK; School of Medicine, Cardiff University, Cardiff, UK

## Abstract

**Introduction:**

Nicotine salt e-liquids are formulated at lower pH to enhance tolerability, but acidity may corrode device components and elevate exposure to inhaled metals. The relationship between e-liquid pH, aerosol metal concentrations, and user health risks has not been systematically characterized.

**Methods:**

Nine refillable e-cigarette (EC) devices were tested at controlled pH values (8.0, 5.1, 4.0, 3.2). Aerosols were analyzed for aluminum, cadmium, chromium, iron, lead, nickel, and tin using Inductively Coupled Plasma Mass Spectrometry (ICP-MS). Risks were assessed using ICH Q3D inhalation permitted daily exposures (PDE) and US Food and Drug Administration excess lifetime cancer risk (ELCR) methodology.

**Results:**

The UK market survey identified pH 4 as representative of typical e-liquid. At pH 4, median aerosol concentrations were Ni 2649 ppb, Cr 20 ppb, and Pb 176 ppb, compared with Ni 28 ppb, Cr 3 ppb, and Pb non-detectable levels at pH 8. Chromium exceeded its inhalation PDE at pH 4, while nickel and lead remained below their respective thresholds; no other measured metals exceeded PDE limits. The mean combined-metals ELCR increased from 47 per 100 000 at pH 8 to 747 per 100 000 at pH 4.

**Conclusions:**

E-liquid pH is a critical determinant of metal emissions and associated risk in open-system EC, while risks remain lower than smoking, exposures at market-representative pH can exceed regulatory thresholds.

**Implications:**

These findings indicate that e-liquid acidity is a key determinant of metal emissions and metals-attributable cancer risk in open-system electronic cigarettes. While risks remain substantially lower than those associated with combustible smoking, exposures at market-representative pH values can exceed established inhalation thresholds for chromium and produce non-trivial excess lifetime cancer risk. Regulatory frameworks may therefore benefit from considering e-liquid pH, device material compatibility, and routine aerosol metals testing across relevant pH ranges to better align product standards with harm-reduction objectives.

## Introduction

Internationally recognized toxicological frameworks, including the International Council for Harmonization (ICH) Q3D guideline, define inhalation permitted daily exposure (PDE) thresholds for elemental impurities and provide a reference point for evaluating the health relevance of metal exposures from inhaled aerosol.^1^ Electronic cigarettes (ECs) are widely used as alternatives to combustible tobacco, offering inhaled nicotine delivery without combustion.^2^ Cigarette smoking remains one of the leading causes of preventable morbidity and mortality worldwide, largely due to exposure to tar, carbon monoxide, and thousands of combustion-related toxicants.^3^ In contrast, ECs heat and aerosolise liquids containing nicotine and flavorings without combustion, substantially reducing exposure to many harmful constituents. An evidence review commissioned by Public Health England concluded that ECs are around 95% less harmful than smoking.^4^ While debates continue regarding the long-term health impact of ECs, their role in harm reduction is now recognized in several national strategies,^4–6^ while WHO does not endorse ECs as a harm reduction tool.^3^

For never-smokers, any incremental exposure to metals, or other toxicants represents an avoidable increase in risk relative to ambient air.^7^ Population-level surveillance in Great Britain indicates that approximately 8% of current adult EC users, around 440 000 people, have never smoked combustible cigarettes, meaning that any exposure to inhaled metals represents an incremental risk above ambient air rather than a reduction from smoking-related harms.^8^ Given that a sizeable proportion of EC users report no history of combustible tobacco use, framing risk in relation to background environmental exposure is essential for public-health relevance.

Open-system ECs allow users to fill a tank or cartridge with an e-liquid of their choice, provide consumers with flexibility and variety compared with closed-system products.^9^ However, the same flexibility introduces variability in emissions and raises concerns regarding toxicant exposure. A consistent finding across the literature is the presence of metals in EC liquids and aerosols, and even in the biospecimens of users.^9–12^ The term “heavy metals” is used here in the conventional toxicological sense to denote elements with known systemic or carcinogenic toxicity (eg, Ni, Cr, Pb, Cd), rather than as a strict chemical classification. These metals originate primarily from heating coils and other components in direct contact with e-liquids.^10^^,^^13^ Studies have directly compared metal concentrations based on device type, demonstrating significant variability in aerosol output.^10^^,^^14^^,^^15^ Among them, nickel and chromium are particularly concerning due to their potential to elicit respiratory irritation, sensitization, and, in certain chemical states, carcinogenic effects.^16^ The toxicological mechanisms of chromium, particularly the hexavalent form, are well-established, linking it to oxidative stress and DNA damage.^17^ Nickel and chromium are principal constituents of the alloys used in heating elements (eg, nichrome, stainless steel), creating a direct mechanistic link between coil composition, corrosion behavior, and aerosol metal output.^10^^,^^11^^,^^18^ Lead, a systemic toxicant with no safe level of exposure,^19^ further elevates concerns regarding cumulative health risk.

The introduction of nicotine salt formulations reshaped the EC market. These formulations reduce the alkalinity of nicotine by adding organic acids,^20^ lowering e-liquid pH and improving tolerability.^21–23^ This allows higher nicotine concentrations to be inhaled with less irritation, increasing appeal among consumers. However, the acidification of e-liquids may promote corrosion of metallic components, enhance the dissolution of metal and increasing transfer into aerosol.^10^^,^^14^ Several studies have reported metals in aerosols from acidified liquids, but the specific role of pH across commonly used devices, and the resulting health risks, has not been systematically evaluated.^24^

Regulatory frameworks highlight the need for quantitative assessment. The International Council for Harmonization (ICH) Q3D guideline specifies inhalation PDE thresholds for elemental impurities,^1^ while the US Food and Drug Administration (FDA) applies an excess lifetime cancer risk (ELCR) approach for carcinogen.^25^ These approaches provide benchmarks for evaluating the toxicological significance of metals detected in EC aerosols. However, few studies have explicitly linked e-liquid acidity to metal release and risk estimates using these regulatory standards.

This study aimed to (1) quantify aerosol metal concentrations across controlled e-liquid pH conditions in open-system electronic cigarettes, (2) evaluate the extent to which observed exposures exceed ICH Q3D inhalation PDE thresholds, (3) estimate metals-attributable ELCR using FDA methodologies, and (4) contextualize laboratory findings against the pH distribution of e-liquids available on the UK market.

## Materials and Methods

Study design and devices. Nine refillable devices common in the UK market were tested: GeekVape Sonder Q, Vaporesso XROS 4, Voopoo Vmate i2, Elf Bar ELFY, Aspire Gotek X, Aspire PockeX, Innokin Endura T18, Aspire K2, and H2 eGO. This ensured heterogeneity in corrosion susceptibility and metal-release profiles. Devices were new and operated at manufacturer appropriate power and coil resistances (see Table S4. Devices and operating characteristics used in the study). Devices were selected to represent the structural and material diversity of UK open-system products. Selection criteria included coil-type variation (nichrome, stainless steel, mesh, integrated wick), airflow design, and power range.

### E-Liquid and Measured pH

A nicotine containing base at 70%–30% PG to VG with about 1% wt/wt nicotine was prepared. A nicotine concentration of ~1% was used to create a best-case formulation with minimal acidity or viscosity effects. This ensured that any increase in metal release reflected pH alone, rather than additional acidity from higher-strength nicotine salt liquids. Lactic acid was added to achieve nominal pH 8, 5, 4, and 3. (see Table S2. Base e-liquid composition). pH was measured using a Mettler-Toledo Seven-Compact S220 pH meter. Measured pH values were 8.0, 5.1, 4.0, and 3.2 for the nominal pH 8, 5, 4, and 3 conditions. Devices were filled with about 2.0 mL and stored for seven days at room temperature to allow liquid hardware contact prior to aerosol collection.

### Aerosol Generation and ICP-MS

Devices were machine vaped (Cerulean, 8-port E-cigarette Testing Instrument) applying an EN ISO 20768:2018 regime (55 mL, 3 s puffs, 30 s interval), 50 puffs per sample.^26^ Aerosol condensate and parallel liquid samples were analyzed for Al, Cd, Cr, Fe, Pb, Ni, and Sn by ICP-MS using multi element calibration, internal standards, blanks and collision or reaction cell mode to mitigate interferences. ICP-MS analysis was conducted on an Agilent 7850 system with autosampler under the following conditions: RF power 1550 W, nebulizer gas flow 1.05 L/min, pump rate 0.10 RPS, spray chamber temperature 2°C, and helium collision gas. External calibration (1–100 ng/mL) was applied with verification at 25 ng/mL. Scandium, iridium, terbium, and indium served as internal standards. Solvent blanks and spiked reference samples were included for quality control. For the metals of interest (Al, Cd, Cr, Fe, Pb, Ni, and Sn), limits of detection ranged from 0.05 to 5.0 μg/g in e-liquids and 0.05 to 0.5 μg/50 puffs in aerosols, with corresponding limits of quantification between 0.06 and 6.0 μg/g for e-liquids and 0.06–0.6 μg/50 puffs for aerosols.

Each device-pH condition was evaluated using a single aerosol collection run comprising 50 puffs generated under the EN ISO 20768:2018 regime. This single-replicate structure reflects the design intent of the study, which was to quantify pH-driven directional changes across devices rather than to characterize absolute variability within devices. To ensure internal consistency, device mass loss per puff, aerosol mass yield, and instrument stability checks were performed before and after each run. Across all device–pH combinations, these routine QC checks demonstrated low operational variability, with puff-to-puff aerosol mass differences remaining within the expected tolerance window of the Cerulean CETI-8 system. Although replicate aerosol collections were not performed, the stability of upstream QC metrics indicates that the observed differences across pH conditions are driven by chemistry rather than procedural variation. Details of the ICP-MS method and validation particulars are presented in Table S5.

### Particle Size Determination

Particle size distribution of aerosols was measured by laser diffraction using a Malvern Spraytec instrument equipped with an inhalation cell, vacuum pump, and flow controller. Devices were operated at defined flow rates (typically 30 or 60 L/min), and aerosols were drawn through the inhalation cell, where particle size distributions were recorded at a data acquisition rate of 100 Hz. Measurements were conducted in rapid mode with a 300 mm lens and analyzed to obtain volume-based size distributions. For each sample, multiple runs were averaged to ensure reproducibility, and background checks were performed prior to analysis to confirm the absence of contamination. Results were expressed as Dv_(10)_, Dv_(50)_, and Dv_(90)_ values in micrometers, with a reference device (50/50 propylene glycol/glycerol formulation) analyzed alongside each batch for quality control. One way ANOVA and Kruskal–Wallis tests were used to evaluate pH effects. An alpha value of less than 0.05 was considered statistically significant (see Table S4: Particle Size (Dv_(10)_, Dv_(50)_, Dv_(90)_) across pH range).

### UK e-Liquid pH Survey

The UK pH survey comprised 142 products comprising open and closed systems with pH measured by bench top meter with temperature compensation. Results of measured pH in UK e-liquids are presented supplementary to this study (see Table S3). Measured levels of UK sourced ECs’ e-liquid (open and closed systems).

### Statistical Analysis

Statistical evaluation focused on assessing the effect of e-liquid pH on aerosol metal concentrations and particle-size metrics. Because metal concentration data were not normally distributed, between-pH comparisons were conducted using Kruskal–Wallis tests. One-way ANOVA was applied to particle-size measurements, where data met the assumptions of the test. Statistical significance was defined as *p* < .05.

### Toxicological Evaluation

ELCR was calculated for metals with inhalation unit risks adopted by FDA Centre for Tobacco Products (CTP)^25^ from Environmental Protection Agency (EPA) Integrated Risk Information System and Californian EPA, namely nickel, chromium, and lead were detected. FDA CTP expresses risk as the daily intake associated with an ELCR of 1 per 100 000 people. The ELCR quantifies the incremental cancer burden attributable to exposure to specific toxicants, above the baseline risk observed in the general population (ie, the additional cancer cases expected relative to ambient-air exposure conditions).We converted measured ppb in condensate to mass per puff, scaled to daily intake using device specific change in mass per puff and assuming a maximum nicotine concertation of 20 mg/mL and a standard daily nicotine intake of 40 mg^25^ and then divided by the risk value to yield ELCR per 100 000.

Daily intake (μg/day) for each metal was computed as:


$$ \mathrm{Mass}\ \mathrm{per}\ \mathrm{puff}\ \left(\mathrm{\mu} \mathrm{g}/\mathrm{puff}\right)\times \mathrm{TPM}\times 40\ \mathrm{mg}\ \mathrm{nicotine}/\mathrm{day}\div 20\ \mathrm{mg}/\mathrm{mL} $$

where the TPM (Total Particulate Matter) represents the device-specific change in liquid mass per puff.


\begin{align*} \mathrm{TPM}=\left(\mathrm{Mass}\ \mathrm{of}\ \mathrm{device}\ \mathrm{before}\hbox{--} \mathrm{Mass}\ \mathrm{of}\ \mathrm{device}\ \mathrm{after}\right)/\\ \mathrm{Number} \mathrm{of}\ \mathrm{puffs} \end{align*}

This approach normalizes exposure across devices with different aerosol generation efficiencies by adjusting for actual mass delivered per puff. The combined ELCR is the sum across included metals measured as part of this research (Table 2).

For chromium, total measured concentrations were conservatively assumed to represent hexavalent chromium [Cr (VI)], consistent with ICH Q3D^1^ practice. This assumption likely overestimates risk, as a proportion of chromium in EC aerosols is expected to be present in the less toxic trivalent [Cr (III)] form. By adopting the Cr (VI) assumption, our analysis biases toward higher risk estimates, prioritizing public health protection and ensuring results are not underestimated. Relative cancer risk to combustible cigarettes was determined through comparison of measured ELCR against that of a reference cigarette (10 000 per 100 000).^25^

PDEs were applied from ICH Q3D^1^ to evaluate non-cancer risk.

## Results

### pH Levels in e-Liquids in the UK Market

The UK e-liquid survey demonstrated that most liquids cluster around pH 4–5 with a median of pH 4.4 and a range of pH 3.4 to pH 8.9 (*n* = 142). Approximately two-thirds of products measured between pH 4.1 and 5.0.Only five of the 142 surveyed liquids (3.5%) measured at or below pH 3.5. Accordingly, the laboratory condition of pH 4 represents a realistic market-representative value, while the measured pH 3.2 condition reflects an extreme lower-tail scenario.

### Aerosol Metal Concentrations by pH and Device

**Figure 1:**
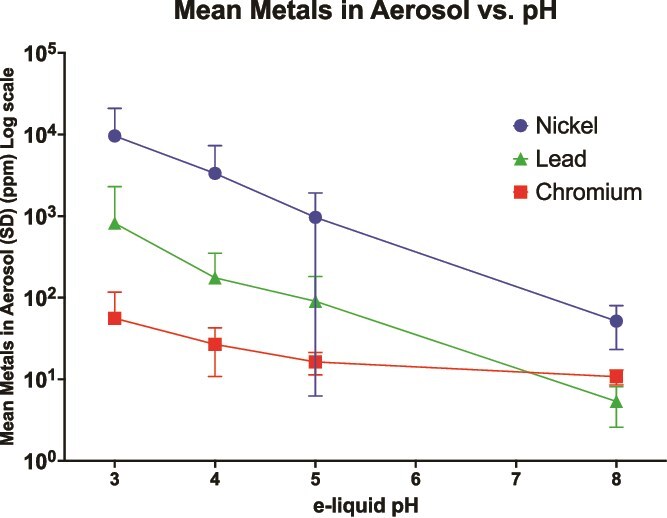
Total aerosol metals versus e-liquid pH, median across devices. Mean concentrations (ppb) of selected metals (nickel, lead, and chromium) in aerosol condensate measured across pH levels 3–8. Bars represent the mean value; error bars represent the standard deviation (SD) across the nine open-system devices tested at each pH condition (*n* = 9).

Median total aerosol metals increased by about 16-fold from about 496 ppb at pH 8 to about 8136 ppb at pH 3 (Table 1; Figure 1). Nickel increased from 27.7 to 7402 ppb, chromium from 2.5 to 20.2 ppb and lead from not detected to 374 ppb. Tin rose from 3.1 to 78.0 ppb, while iron exhibited a non-monotonic pattern across pH conditions (Table 1).

**Table 1:** Median aerosol metal concentrations in ppb by e-liquid pH.

pH	Al Median (Q1–Q3)	Cd Median (Q1–Q3)	Cr Median (Q1–Q3)	Fe Median (Q1–Q3)	Pb Median (Q1–Q3)	Ni Median (Q1–Q3)	Sn Median (Q1–Q3)	Total (sum of medians)
pH 8	780.7 (726.2–1181.1)	0.0 (0.0–0.0)	10.7 (10.0–12.4)	173.2 (129.2–486.6)	4.5 (3.5–5.3)	44.5 (37.0–72.6)	6.0 (5.1–6.5)	1019.6
pH 5	521.8 (467.8–1128.2)	0.0 (0.0–0.0)	15.0 (13.2–21.1)	163.3 (118.1–191.6)	71.9 (22.4–93.2)	774.8 (393.8–888.8)	30.3 (19.2–36.1)	1577.1
pH 4	821.4 (612.6–1092.3)	0.0 (0.0–0.0)	20.4 (17.2–27.9)	323.9 (281.5–422.1)	119.5 (59.3–232.9)	1509.2 (971.1–3145.7)	32.6 (27.7–51.2)	2826.9
pH 3	672.0 (520.6–1022.1)	0.0 (0.0–0.0)	34.3 (26.0–54.5)	405.7 (310.9–716.7)	251.6 (20.9–761.2)	4947.6 (3846.8–10139.6)	73.4 (17.2–95.3)	6384.6

Values are expressed in parts per billion (ppb) and presented as Median (Q1–Q3) across nine devices at each measured pH condition. Values below the analytical limit of detection were imputed as 0.0 ppb. Total represents the sum of individual metal medians for each pH condition.

Al, aluminum; Cd, cadmium; Cr, chromium; Fe, iron; Pb, lead; Ni, nickel; Sn, tin.

**Figure 2:**
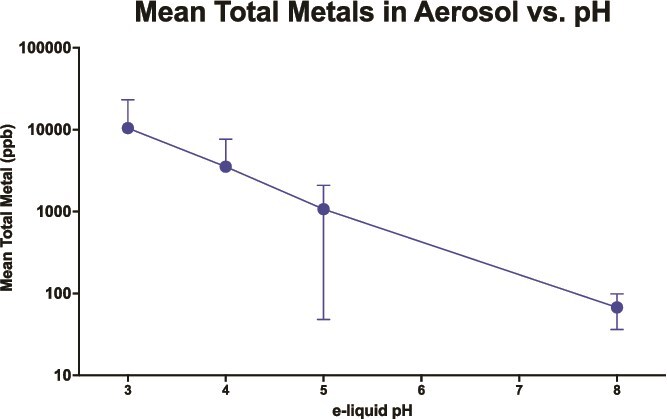
Median aerosol nickel, chromium and lead versus pH. Mean total metal concentration (sum of all measured analytes in ppb) in aerosol condensate as a function of e-liquid pH. Bars represent the mean of the nine devices; error bars represent the standard deviation (SD), illustrating the variability in total metal output driven by hardware differences at each pH level.

At pH 4, median aerosol concentrations were Ni 2649 ppb, Cr 20.3 ppb, and Pb 176 ppb with Sn 23.6 ppb and Fe 106 ppb. Several devices produced very high nickel at pH 4 for example greater than 12 000 ppb in Aspire Gotek X and about 6600 ppb in Voopoo Vmate i2. Chromium rose across devices with a group mean of about 20 ppb and a median of about 13 ppb (Figure 2). Non-parametric testing (Kruskal–Wallis) demonstrated statistically significant differences across pH conditions for nickel, chromium, and lead (*p* < .05).

### Excess Lifetime Cancer Risk by pH and Device

**Figure 3:**
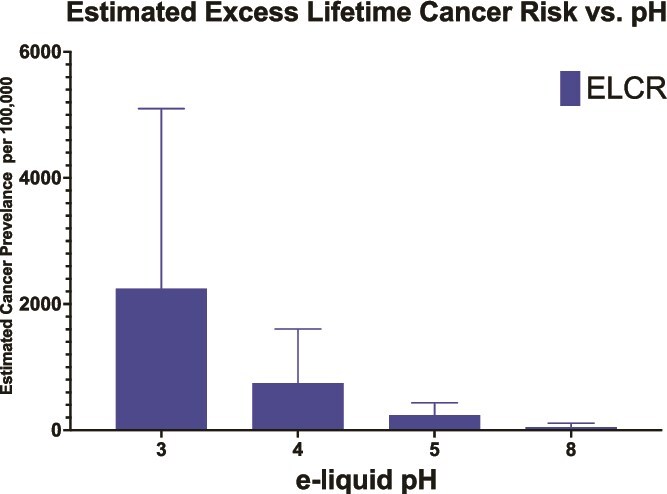
Combined metals excess lifetime cancer risk per 100 000 versus pH. Estimated excess lifetime cancer risk (ELCR) associated with daily e-cigarette use across the pH range 3–8. Calculations assume conservative hexavalent chromium toxicity. Bars represent the mean ELCR calculated for the nine devices; error bars represent the standard deviation (SD). The horizontal dashed line indicates the acceptable risk threshold 1 in 100 000.

Combined metals ELCR showed strong pH correlation and marked device variability (Table 2; Figure 3). At pH 4 the mean ELCR across devices was 747 per 100 000 with device values ranging from 66 to 2668 per 100 000. At measured pH 3.2 the mean ELCR was 2245 per 100 000 with device values from 235 to 8277 per 100 000.

**Table 2:** Device specific combined metals ELCR per 100 000 by pH and % risk of smoking.

Device	ELCR pH 8	ELCR pH 5	ELCR pH 4	ELCR pH 3
Device A	57	558	538	1540
Device B	20	157	548	235
Device C	0	47	252	409
Device D	17	62	66	458
Device E	3	207	1272	1865
Device F	27	150	218	450
Device G	194	532	414	4727
Device H	60	182	2668	8277
Device I	13	108	407	1764
Mean	43.4	222.6	709.2	2080.6
% Risk of Smoking	0.43%	2.23%	7.09%	20.81%

ELCR denotes excess lifetime cancer risk expressed per 100 000 users and calculated for nickel, chromium, and lead using inhalation unit risks adopted by FDA CTP. Device-specific values reflect metals-only cancer risk and are normalized to a daily nicotine intake of 40 mg. The cigarette comparator assumes an approximate total ELCR of 10 000 per 100 000 users. Values below the limit of detection were treated as zero. The “% risk of smoking” comparison relates exclusively to metals and does not include contributions from aldehydes, tobacco-specific nitrosamines, or other carcinogenic compounds.

At pH 8 and pH 5, means were 47 and 237 per 100 000, respectively. When expressed as a fraction of a typical cigarette risk level of 10 000 per 100 000 for all carcinogens combined, the metals only ELCR corresponds to 0.5% at pH 8, 2.4% at pH 5, 7.5% at pH 4 and 22.5% at pH 3 on average across devices (Table 2. Device specific combined metals ELCR per 100 000 by pH, Figure 3. Combined metals ELCR per 100 000 versus pH). For never-smokers, the combined-metals ELCR at pH 4 represents a 3–8× increase relative to typical UK ambient-air metal-related cancer risks, depending on regional background concentrations.

### Permissible Daily Exposure by pH and Device

Metal intakes were compared against inhalation PDE thresholds specified in ICH Q3D. At pH 4, median metal exposures corresponded to 0.37× PDE for nickel, 1.1× PDE for chromium, and 0.05× PDE for lead. Chromium therefore exceeded its inhalation PDE on a median basis at pH 4, with exceedance becoming more pronounced at pH 3. Nickel and lead remained below their respective PDE thresholds at the median, and no other measured metals exceeded inhalation PDE limits. Median exposures relative to inhalation PDE thresholds across pH conditions are summarized in Table 3.

**Table 3:** Device-aggregated multiples of ICH Q3D inhalation PDE (×PDE) by pH, calculated using TPM-derived aerosol mass and device-specific puffs per Day.

Metal (PDE, μg/day)	pH 8 median (Q1–Q3)	pH 5 median (Q1–Q3)	pH 4 median (Q1–Q3)	pH 3 median (Q1–Q3)
Nickel (6.0)	0.33 (0.28–0.54)	5.77 (2.93–6.62)	11.2 (7.23–23.4)	36.8 (28.6–75.5)
Chromium (3.0)	0.16 (0.15–0.18)	0.22 (0.20–0.31)	0.30 (0.26–0.42)	0.51 (0.39–0.81)
Lead (5.0)	0.040 (0.031–0.047)	0.64 (0.20–0.83)	1.07 (0.53–2.08)	2.25 (0.19–6.80)
Cadmium (3.0)	ND	ND	ND	ND
Tin (60.0)	0.004 (0.004–0.005)	0.023 (0.014–0.027)	0.024 (0.021–0.038)	0.055 (0.013–0.071)
ΣPDE (screening-level non-cancer hazard index)	0.53 (0.47–0.77)	6.65 (3.34–7.79)	12.6 (8.04–25.9)	39.6 (29.2–83.2)

Values are expressed as multiples of the ICH Q3D inhalation Permissible Daily Exposure (PDE). Aerosol metal concentrations were treated as mass-based concentrations (ppb ≡ μg/g aerosol). Device-specific total particulate matter (TPM) was determined gravimetrically as the change in device mass during a standardized 50-puff aerosol generation run conducted under EN ISO 20768:2018 conditions. Mean aerosol mass per puff was calculated as TPM/50 and multiplied by the device-specific number of puffs per day (Table S1), derived to achieve a total daily nicotine intake of 40 mg assuming a maximum e-liquid nicotine concentration of 20 mg/mL, consistent with UK regulatory limits and U.S. FDA Centre for Tobacco Products exposure-assessment conventions. Resulting daily metal intakes (μg/day) were normalized to the relevant ICH Q3D inhalation PDE to yield ×PDE values.

The summed ×PDE (ΣPDE) represents a screening-level non-cancer hazard index, calculated as the sum of individual metal PDE ratios, and assumes additive effects for conservative risk characterization. It does not represent a mechanistic cumulative toxicity model and is distinct from excess lifetime cancer risk (ELCR) estimates.

While nickel remained below its PDE at the median, chromium (applying an assumption of hexavalent)^1^ exceeded the PDE on a median basis at pH 4 and more so at pH 3. Chromium was conservatively assumed to be present entirely as hexavalent chromium; speciation analysis would be required to confirm this assumption.

### Aerosol Particle Size by pH

Particle size distributions were stable across all pH conditions. Median Dv50 values were approximately 0.6 μm for all devices and pH levels, with no systematic trend observed as a function of e-liquid acidity. Statistical analysis showed no significant effect of pH on aerosol particle size metrics (Dv10, Dv50, Dv90), indicating that observed differences in metal concentrations were not attributable to changes in aerosol physics.

## Discussion

This study found that lower e-liquid pH substantially increases aerosol metal concentrations and elevates metals-attributable cancer risk in open-system electronic cigarettes. The effect was strongest for nickel and clearly present for chromium and lead, while cadmium remained negligible. Because particle size did not change meaningfully with pH,^27^ the driver is best explained by acid-related corrosion and dissolution of metals and breakdown of protective oxides on coils and other e-liquid contact parts.^18^^,^^28^

The UK market survey anchors these findings in real-world relevance: most liquids clustered around pH 4–5, with a median of 4.4. The pH 4 condition therefore reflects a realistic median for current UK products, while pH 3.2 represents a lower-tail scenario. At pH 4, the median combined metals ELCR was 538 per 100 000, and the mean across devices was 747 per 100 000. Device-level ELCR varied by more than an order of magnitude (66-2668 per 100 000), underscoring the importance of materials, design, and compatibility between liquids and hardware.

Interpretation of these ELCR values requires context. FDA’s Center for Tobacco Products applies ELCR in regulatory reviews, with cigarette smoking estimated at 10000 per 100 000 and authorized EC products reporting means around 118 per 100 000. Our metals-only ELCR values therefore fall well below cigarette risks,^29^ but higher than published estimates for authorized closed-system products.^24^ Importantly, our values reflect only metals and do not account for aldehydes, nitrosamines, or other, all of which contribute to cumulative toxicological burden.^9^^,^^30^ Chromium was conservatively treated as hexavalent, which biases estimates toward overstatement, but is consistent with regulatory practice in the absence of speciation data.^1^^,^^31^

Unlike smokers transitioning to ECs, never-smokers do not benefit in a reduction from a higher baseline exposure risk; instead, they incur incremental exposure above ambient environmental levels. When compared against non-exposure of chromium and nickel, the ELCR values observed at pH 4 represent several-fold increases in lifetime cancer risk relative to non-use. Although absolute risks remain below those associated with cigarette smoking, the use of low-pH liquids by never-smokers introduces avoidable toxicant exposure that would not occur under normal ambient conditions. This distinction is critical for regulatory interpretation, particularly as a meaningful proportion of EC users report no prior history of combustible tobacco use.^8^

These results highlight that the harm-reduction profile of EC is not fixed but conditional. While risks remain far lower than those from smoking, open-system devices used with low-pH nicotine salt liquids may expose users to levels of metals that surpass regulatory thresholds.^24^ In particular, chromium exceeded its ICH Q3D inhalation PDE at pH 4, and nickel reached over one-third of its PDE. Thus, pH and device design emerge as critical determinants of safety within the category.^10^^,^^14^

From a regulatory and policy perspective, these findings carry several implications. Current frameworks (FDA PMTA, UK MHRA, EU TPD) do not set explicit limits on e-liquid acidity. Our data suggest that incorporating pH guidance, mandating aerosol metals testing across relevant pH ranges, and incentivizing the use of corrosion-resistant materials could substantially reduce harms. Policies should also consider that nicotine salts, which lower pH, improve product tolerability^32^ and may support smoking cessation. Striking a balance between maintaining product appeal for switching and minimizing toxicant exposure will be central to regulatory decision-making.

Strengths of this work include use of measured pH values, analysis of seven metals by validated ICP-MS with quality assurance, nine market-relevant devices, and a UK e-liquid survey to contextualize laboratory findings. Limitations include lack of chromium speciation, testing of a single puffing regimen, and use of lactic acid only to adjust pH. The seven-day e-liquid and device exposure time reflects early-use corrosion behavior but does not capture progressive aging, nor account for repeated heat-cycles expected during normal use.^33^ Future research should extend to multiple acid systems, examine corrosion-resistant coatings and alloys, and integrate non-metal toxicants into cumulative risk models.

## Conclusions

The relative safety of open-system ECs is strongly dependent on e-liquid pH. At higher pH (>5), heavy metal risks appear negligible; at lower pH (<5), particularly around the UK market median of 4.4, exposures to nickel and chromium rise substantially, with chromium exceeding its PDE and combined-metals ELCR reaching levels regulators may judge non-trivial. Although these risks remain below those from smoking, they are elevated relative to authorized products and highlight the conditional nature of EC harm reduction.

Maintaining formulations above pH 5 could substantially lower metals exposure and associated cancer risk, improving the risk profile of open-system products. Regulatory standards should therefore consider mandatory aerosol metals testing across pH conditions, incorporate explicit pH guidance, and incentivize corrosion-resistant materials and designs.^24^ These steps would align product regulation more closely with public health objectives and help ensure that harm reduction is realized in practice.

## Supplementary Material

Supplementary_Tables_Clean_V3_ntag016

Supplementary_-_E-Liquid_Survey_ntag016

## Data Availability

Data, raw ICP-MS outputs and analysis worksheets are available from the corresponding author on reasonable request.

## Patient and public involvement

No patient or public involvement.
