# Supplementary material for: Influence of e-Liquid pH on Heavy Metal Emissions in Open-System Electronic Cigarette Aerosols and Associated Health Risks
Source: Nicotine Tob Res. 2026 Mar 27;28(7):1200–7. doi: 10.1093/ntr/ntag016 (PMC13286629; doi:10.1093/ntr/ntag016)
Supplement: Supplementary_Tables_Clean_V3_ntag016 [file supplementary_tables_clean_v3_ntag016.docx]

**Supplementary Tables**

**Table S1.** Devices and operating characteristics used in the study

| Manufacturer | Brand | Format | Power (W) | Coil (Ω) | TPM (mg) | Puffs per day |
| --- | --- | --- | --- | --- | --- | --- |
| GeekVape | Sonder Q | Pod | 11 to 16 | 0.8 | 19.9 | 112 |
| Vaporesso | XROS 4 Mini | Pod | 11 to 16 | 0.4 | 31.6 | 71 |
| Voopoo | Vmate i2 | Pod | 12 to 17 | 0.7 | 16.5 | 135 |
| Elf Bar | ELFY | Pod | 9 to 15 | 0.8 | 22.5 | 99 |
| Aspire | Gotek X | Pod | 13 to 16 | 0.8 | 13.9 | 161 |
| Aspire | PockeX | Tank | 18 to 23 | 0.6 | 10.2 | 219 |
| Innokin | Endura T18 | Tank | 13.5 | 1.5 | 18.6 | 120 |
| Aspire | K2 | Tank | 12 | 1.6 | 15.2 | 147 |
| Unknown | H2 (eGO) | Tank | 11 | 2.2 | 4.0 | 559 |

***Note****: Device operating parameters (power range and coil resistance) were taken from manufacturer specifications where available. Total particulate matter (TPM) represents the device-specific change in mass during a standardised 50-puff aerosol generation run conducted under EN ISO 20768:2018 conditions and was determined gravimetrically by weighing devices before and after vaping. The derived number of puffs per day was calculated on a device-specific basis as the number of puffs required to achieve a total daily nicotine intake of 40 mg, assuming a maximum e-liquid nicotine concentration of 20 mg/mL in line with UK regulatory limits. This approach follows U.S. FDA Centre for Tobacco Products exposure-assessment conventions and was used to scale per-puff metal emissions to estimated daily exposure. The H2 (eGO) device was sourced from Amazon.co.uk and manufacturer details were not available. Devices include both pod and tank formats representative of products commonly used in the UK market.*

**Table S2.** Base e-liquid composition

| Ingredients | Batch Number | E-liquid 70/30 (g/100 g) |
| --- | --- | --- |
| Propylene glycol | 2379648 (Fisher) | 67.90 ± 0.50 |
| Glycerol | 2488108 (Fisher) | 29.10 ± 0.50 |
| Water | N/A | 1.00 ± 0.10 |
| Ethanol | 2400707 (Fisher) | 1.00 ± 0.05 |
| Nicotine | A0450530 (Thermo Scientific) | 1.00 ± 0.05 |
| Lactic Acid | (Reagent grade) | Sufficient to adjust pH |

***Note****: The 70/30 PG:VG formulation was prepared following the AFNOR XP D90-300 benchmark for e-liquid composition to ensure consistency of matrix properties across pH conditions. Reagent-grade inputs were used for all components, and lactic acid was added solely to achieve the required pH adjustments.*

**Table S3** Measured levels of UK sourced ECs’ e-liquid (open and closed systems)

| Brand | Flavour | Nicotine (mg/mL) | pH |
| --- | --- | --- | --- |
| Vuse | Blackcurrant Ice | 18 | 8.9 |
| Elf Bar | Strawberry Raspberry Cherry Ice | 20 | 8.6 |
| Superior Vapour | Forest Fruits | 3 | 8.4 |
| ELFIQ | Rhubarb Snow | 10 | 7.3 |
| Pod Salt | Strawberry Marshmallow | 20 | 6.7 |
| Heaven Haze | Peanut Butter Chocolate Fudge Ice Cream | 3 | 6.7 |
| Jucce Bar | Lemon Lime | 12 | 6.6 |
| SKE | Cherry Ice | 20 | 6.2 |
| Hayati Pro Ultra 15000 | Cherry Ice | 20 | 5.7 |
| Elf Bar | Watermelon | 20 | 5.7 |
| Vaporesso | N/A | 20 | 5.2 |
| SKE | Fizzy Cherry | 20 | 5.1 |
| Hayati Twist | Strawberry Raspberry Ice | 20 | 5.0 |
| Lost Mary | Blue Razz Ice | 20 | 5.0 |
| Crystal Ultimate | Energy Blast | 20 | 5.0 |
| JNR Mega Box | Fizzy Cherry Cola | 20 | 4.9 |
| SKE | Strawberry Burst | 20 | 4.9 |
| Elux | Lime Lemon | 20 | 4.8 |
| FlerBar | Pink Watermelon | 20 | 4.8 |
| Jucce Bar | Pink Lemonade | 12 | 4.8 |
| OG Super Smash | VIM Energy Ice | 20 | 4.8 |
| IVG | Strawberry Ice | 20 | 4.8 |
| Crystal Clear | Strawberry Watermelon Bubble gum | 20 | 4.8 |
| ELFIQ | Apple Peach | 10 | 4.8 |
| RAD | Watermelon Breaker | 20 | 4.8 |
| Pixl | Hawaiian Oasis | 20 | 4.8 |
| Flavaah | Blue Razz Berry | 20 | 4.8 |
| Bloody Bar | Strawberry Watermelon B-Gum | 20 | 4.8 |
| Lost Mary | Pineapple Ice | 20 | 4.8 |
| Veev | Lemon Lime | 18 | 4.8 |
| Jucce | Tobacco | 12 | 4.8 |
| ELFIQ | Spearmint | 10 | 4.7 |
| S-Elf | Fuji Apple | 20 | 4.7 |
| Gold Bar | Strawberry Watermelon | 20 | 4.7 |
| Kingston | Tutti Fruitti | 20 | 4.7 |
| IVG Salt | Fresh Lemonade | 10 | 4.7 |
| Elf Bar | Cherry | 20 | 4.6 |
| Crystal Clear | Xtreme Sour Apple | 20 | 4.6 |
| ELFIQ | Watermelon | 20 | 4.6 |
| IVG | Triple Melon | 20 | 4.6 |
| Jucce Bar | Lemon Lime | 12 | 4.6 |
| Pixl | Lemon & Lime | 20 | 4.6 |
| Hayati | Fresh Mint | 20 | 4.6 |
| Elux | Blueberry Sour Raspberry | 20 | 4.6 |
| Lost Mary | Blue Razz Cherry | 20 | 4.6 |
| Firerose | Blue Razz Lemonade | 20 | 4.6 |
| Sad Boy | Shamrock Cookie | 20 | 4.6 |
| Crystal Ultimate | Blueberry, Cherry & Cranberry | 20 | 4.6 |
| Ultimate Bar XL | Gummy Bear | 20 | 4.6 |
| Airis Max | Lush Ice | 20 | 4.5 |
| Jucce Bar | Fruit Twist | 12 | 4.5 |
| Major Flavour | Bettle Juice | 20 | 4.5 |
| IVG | Watermelon Ice | 20 | 4.5 |
| ELFIQ Nic Salts | Triple Melon | 10 | 4.5 |
| Flavaah | Blue Razz Berry | 20 | 4.5 |
| Fecba | Gummy Bear | 20 | 4.5 |
| Hayati | Hubba Bubba | 20 | 4.5 |
| Blu Bar | Watermelon Ice | 20 | 4.5 |
| Bloody Bar | Lemon Ice | 20 | 4.5 |
| TECC | Apple Pie & Custard | 10 | 4.5 |
| Pyne Pod | Pineapple Ice | 20 | 4.5 |
| Lost Mary | Pink Lemonade | 20 | 4.5 |
| Lost Mary | Blackcurrant Apple | 20 | 4.5 |
| Hayati | Cherry Ice | 20 | 4.5 |
| Prime+ | Gummy Bear | 20 | 4.4 |
| Elux | Jungle Juice | 20 | 4.4 |
| Major Flavour | Desserts | 20 | 4.4 |
| ELFIQ Nic Salts | Tobacco | 10 | 4.4 |
| ELFIQ Nic Salts | Banana Ice | 20 | 4.4 |
| ELFIQ | Blueberry Razz Lemonade | 10 | 4.4 |
| Crown Bar Al Fakher | Two apple | 20 | 4.4 |
| Blu Bar | Banana Ice | 20 | 4.4 |
| Vuse | Mint Ice | 20 | 4.4 |
| SKE | Cotton Ice | 20 | 4.4 |
| Elf Bar | Unnamed | 20 | 4.4 |
| Crystal Pro | Pineapple Ice | 20 | 4.4 |
| Bar Juice | Strawberry Kiwi | 20 | 4.4 |
| SKE | White Peach Razz | 20 | 4.4 |
| Vuse | Strawberry Kiwi | 20 | 4.4 |
| VLTZ | Sour Apple | 16 | 4.4 |
| ELFIQ | Peach Ice | 10 | 4.4 |
| McKesse MKbar | Gummy Bear | 20 | 4.3 |
| Pod Salt | Peanut Butter Banana Granola | 20 | 4.3 |
| Blu Liquid | Menthol | 18 | 4.3 |
| IVG | Strawberry Mint Menthol Mojito | 20 | 4.3 |
| VEEV now | Watermelon | 18 | 4.3 |
| Hayati Twist | Strawberry Kiwi | 20 | 4.3 |
| SKE | Blueberry Raspberries | 20 | 4.3 |
| Bloody Bar | Lemon Ice / Pineapple Ice | 20 | 4.3 |
| Jucce Bar | Lemon Peach Passion Fruit | 12 | 4.3 |
| TECC | Rainbow Slush | 10 | 4.3 |
| Superior Vapour | Blueberry Ice | 3 | 4.3 |
| SKE | Cola Ice | 20 | 4.3 |
| SKE | Sour Apple | 20 | 4.3 |
| Crystal Ultimate | Energy Blast | 20 | 4.3 |
| EDGE | Strawberry | 6 | 4.3 |
| Lost Mary | Triple Mango | 20 | 4.3 |
| IVG | Strawberry Watermelon | 20 | 4.2 |
| Fruitz | Lime Lemonade | 20 | 4.2 |
| ELFIQ | Blueberry | 10 | 4.2 |
| Lost Mary | Kiwi Passion Fruit Guava | 20 | 4.2 |
| Elux | Watermelon Ice | 20 | 4.2 |
| Crystal Pro | Banana Ice | 20 | 4.2 |
| Elux | Apple Peach | 20 | 4.2 |
| ENE | Watermelon Ice | 20 | 4.2 |
| ELFIQ | Elf bull Ice | 10 | 4.1 |
| PIXL | Juicy Peach | 20 | 4.1 |
| Totally Wicked | Green Apple | 14 | 4.1 |
| Pixl | Spearmint | 20 | 4.0 |
| Bar Juice | Cream Tobacco | 20 | 4.0 |
| SKE | Watermelon Strawberry | 20 | 4.0 |
| Elf Bar | Strawberry Raspberry Cherry Ice | 20 | 4.0 |
| Lost Mary | Triple Mango | 20 | 4.0 |
| Vapengin | Fizzy Guava | 20 | 4.0 |
| Bloody Bar | Strawberry Kiwi | 20 | 3.9 |
| Elf Bar | Sour Pineapple Ice | 20 | 3.9 |
| Pyne Pod | Watermelon Ice | 20 | 3.9 |
| BAR Salts | Pink Lemonade | 20 | 3.8 |
| Hayati Pro Max | Skittles | 20 | 3.8 |
| Hayati Pro Max | Blueberry Raspberry | 20 | 3.8 |
| Jucce Bar | Watermelon Ice | 12 | 3.8 |
| Jucce Bar | Kiwi Guava Passion Fruit | 12 | 3.8 |
| Supbliss | Gummy Bear | 20 | 3.7 |
| Fruitz | Sweet Grape | 20 | 3.7 |
| ELFIQ | Cherry | 10 | 3.7 |
| RAD | Candy Kahuna | 20 | 3.7 |
| BAR | Strawberry Ice | 20 | 3.7 |
| BAR Salts | Blue Sour Raspberry | 20 | 3.7 |
| Vaporesso Coss | Double Apple | 20 | 3.7 |
| SKE | Fizzy Cherry | 20 | 3.7 |
| Blu Bar | Kiwi Passionfruit | 20 | 3.7 |
| Blu Bar | Pineapple | 20 | 3.6 |
| Fruitz | Mango Ice | 20 | 3.6 |
| RAD | Bubble Billow | 20 | 3.6 |
| Pixl | Pineapple Ice | 20 | 3.6 |
| Sad Boy | Custard Cookie | 20 | 3.6 |
| Elf Bar | Peach Ice | 20 | 3.6 |
| Elf Bar | Strawberry Snow | 20 | 3.5 |
| Elf Bar | Strawberry Raspberry Cherry Ice | 20 | 3.5 |
| Jucce Bar | Pink Lemonade | 12 | 3.4 |
| Titan | Pineapple Ice | 20 | 3.4 |
| SKE | Watermelon Ice | 20 | 3.4 |
| Min | — | 3 | 3.4 |
| Max | — | 20 | 8.9 |
| Median | — | 20 | 4.4 |

**Note**: *Includes 142 UK-market e-liquids from both open and closed systems. pH values reflect products “as sold,” measured using calibrated instrumentation. Nicotine concentration reported per label claim.*

**Table S4:** Particle Size (Dv_(10)_, Dv_(50)_, Dv_(90)_) across pH range

|  | **Replicate 1** | | | **Replicate 2** | | | **Replicate 3** | | | **Average** | | |
| --- | --- | --- | --- | --- | --- | --- | --- | --- | --- | --- | --- | --- |
| pH | Dv(10) µm | Dv(50) µm | Dv(90) µm | Dv(10) µm | Dv(50) µm | Dv(90) µm | Dv(10) µm | Dv(50) µm | Dv(90) µm | Dv(10) µm | **Dv(50) µm** | Dv(90) µm |
| 3 | 0.27 | 0.39 | 0.57 | 0.25 | 0.39 | 0.61 | 0.26 | 0.38 | 0.56 | 0.26 | **0.39** | 0.58 |
| 4 | 0.28 | 0.38 | 0.51 | 0.22 | 0.38 | 0.64 | 0.23 | 0.38 | 0.62 | 0.25 | **0.38** | 0.59 |
| 5 | 0.23 | 0.39 | 0.64 | 0.26 | 0.41 | 0.64 | 0.21 | 0.39 | 0.72 | 0.23 | **0.40** | 0.67 |
| 8 | 0.21 | 0.42 | 0.82 | 0.25 | 0.39 | 0.59 | 0.23 | 0.39 | 0.68 | 0.23 | **0.40** | 0.70 |

**Note**: *Dv(10), Dv(50) and Dv(90) values represent triplicate particle-size measurements for each pH condition using laser diffraction; averages are arithmetic means of replicates.*

**Table S5:** ICP-MS method parameters and validation data

| **Parameter** | **Method Specification / Validation Outcome** |
| --- | --- |
| Accreditation | ISO/IEC 17025 validated method (TM 11.129.1), accredited by UKAS |
| Instrument | Agilent 7850 ICP-MS with helium collision cell |
| RF Power | 1550 W |
| Carrier Gas Flow | Helium collision mode (validated for Cr, Fe, Ni, Pb, Al) |
| Spray Chamber Temperature | 2 °C (stability-controlled) |
| Interface Cones | Platinum sampler and skimmer cones |
| Diluent / Reagents | Trace-metal grade nitric acid (HNO₃) and methanol (MeOH) |
| Sample Preparation | Direct dilution of condensate in validated 2% HNO₃ matrix |
| Calibration Range | Multi-point calibration, linearity R ≥ 0.999 for all analytes |
| LOD / LOQ Determination | Derived from background-equivalent concentration using S/N ratios of 3 (LOD) and 5 (LOQ), confirmed with replicate low-level standards (Section 4.2) |
| LOD (Aerosol) | 0.02–0.2 µg/50 puffs (analyte-dependent) |
| LOQ (Aerosol) | 0.06–0.6 µg/50 puffs |
| Accuracy / Recovery | Ni: 100.5%; Cr: 102.7%; other analytes 96–110% |
| Precision (Repeatability) | %RSD typically < 8% across analytes |
| Specificity / Interference Testing | No significant spectral interferences: helium collision mode validated to suppress polyatomic ions |
| Stability | Signal drift < 5% over validated 5-hour sequence |
| Quality Controls | Internal standards, calibration verification, oxide ratio checks, doubly charged species checks, sensitivity acceptance criteria |
| Recovery for Key Metals (Ni, Cr) | Within 100 ± 10% of expected values (spiked QC samples) |
| Method Use | Validated for trace-metal quantification in e-liquid and aerosol matrices |

Sample Preparation & Reagents: Aerosol condensate samples were prepared by direct dilution, consistent with the ISO/IEC 17025–validated internal test method TM 11.129.1 method to minimise contamination risk and prevent volatile element loss. Samples were diluted into a matrix of 5% nitric acid (HNO₃) and 10% methanol, with dilution factors matched to the validated method (35× for aerosol condensate; 200× for liquid standards). Calibration standards were prepared in the same matrix to ensure compatibility with the PG/VG sample matrix.

**Note**: *ICP-MS operating conditions, validation criteria, and quality-control procedures reflect the ISO/IEC 17025-validated method used for trace-metal quantification in both e-liquid and aerosol condensate. Method performance (LOD/LOQ, accuracy, precision, and interference suppression) was established using matrix-matched standards, internal standards, and spiked QC samples. All parameters listed represent the certified laboratory conditions under which all study samples were analysed.*
