# Supplementary material for: Influence of e-Liquid pH on Heavy Metal Emissions in Open-System Electronic Cigarette Aerosols and Associated Health Risks
Source: Nicotine Tob Res. 2026 Mar 27;28(7):1200–7. doi: 10.1093/ntr/ntag016 (PMC13286629; doi:10.1093/ntr/ntag016)
Supplement: Supplementary_-_E-Liquid_Survey_ntag016 [file supplementary_-_e-liquid_survey_ntag016.docx]

**Supplementary Methods: UK E-Liquid Market Survey**

A structured UK market survey was undertaken to characterise the real-world distribution of e-liquid pH values and to contextualise the controlled pH conditions used in the laboratory phase of this study. The survey was engineered to reflect the acquisition pathway of an ordinary UK consumer while capturing the breadth of formulations available across reputable online retailers.

Product identification and sampling logic

Products were identified through a standardised Google search using the term “e-liquid,” a typical search behaviour for UK consumers. The search consistently yielded high-volume, reputable UK retailers with visible trading histories and established compliance statements. From this pool, products were selected at random without applying filters for nicotine strength, brand, formulation type, or device category. This ensured broad representation across the market rather than a curated or manufacturer-biased dataset.

Regulatory context and assumptions

All items were sourced exclusively from UK-based vendors operating under the Tobacco and Related Products Regulations (TRPR) 2016. Given their lawful retail availability, products were assumed compliant with TRPR requirements relating to nicotine limits, labelling, and emissions testing. This assumption reflects the regulatory environment that governs actual consumer purchasing behaviour.

Product diversity and market coverage

The final dataset comprised 144 unique products, spanning:

- bottled freebase and nicotine-salt e-liquids,
- short-fill formulations, and
- prefilled pods from closed-system devices (with e-liquid extracted immediately prior to analysis).

These products reflected a wide spread of PG/VG ratios, nicotine strengths (0–20 mg/mL), manufacturer types (legacy brands and newer entrants), and flavour categories.

Shipping, receipt, and traceability

All products were shipped directly from the retailers to the Inter Scientific laboratory at Unit 29, Compass West Industrial Estate, Liverpool, UK. Upon receipt, each product was logged, assigned a unique anonymised survey ID, and stored under controlled ambient conditions until measurement. Labelling metadata, including batch codes, declared nicotine strength, and manufacturer information, were recorded to support traceability and stratified analysis.

Laboratory environment and accreditation

All measurements were conducted at Inter Scientific within a UKAS-accredited laboratory operating under ISO/IEC 17025:2017. This accreditation covers competence in analytical measurement, equipment calibration, and quality management systems, ensuring that survey outputs align with global standards for laboratory reliability and reproducibility.

Sample handling and preparation

For bottled products, approximately 2 mL of e-liquid was transferred into clean polypropylene vials for analysis. For pod-based products, liquid was extracted using single-use low-retention pipettes to avoid cross-contamination. No further preparation was required, reflecting the objective of capturing pH “as sold”.

pH measurement protocol

pH values were measured using a Mettler-Toledo Seven-Compact S220 pH meter equipped with automatic temperature compensation and an LE438 probe. The instrument was calibrated at the start of each measurement session using UKAS-traceable buffer standards at pH 4.00, 7.00, and 10.00. Each product was measured in triplicate, with the probe rinsed and dried between samples. Triplicate measurements demonstrated minimal intra-sample variation (<0.05 pH units), and mean values were used for analysis.

Purpose of the dataset

The objective of the survey was not to characterise chemical composition beyond pH, but to provide a market-representative benchmark against which laboratory pH conditions (8.0, 5.1, 4.0, 3.2) could be contextualised. The dataset therefore offers an operational snapshot of the UK e-liquid market as encountered by consumers, strengthening the external validity of the study’s findings.
